# Psychological and Physical Health Outcomes Associated with Gender-Affirming Medical Care for Transgender and Gender-Diverse Youth: A Critical Review

**DOI:** 10.3390/healthcare13141659

**Published:** 2025-07-10

**Authors:** Terri A. Croteau, Jan Gelech, Melanie A. Morrison, Todd G. Morrison

**Affiliations:** Department of Psychology and Health Studies, University of Saskatchewan, Saskatoon, SK S7N 5A5, Canada; jan.gelech@usask.ca (J.G.); melanie.morrison@usask.ca (M.A.M.); todd.morrison@usask.ca (T.G.M.)

**Keywords:** gender-affirming medical care, transgender and gender diverse, youth, mental health, physical health, minority stress

## Abstract

**Introduction:** Access of transgender and gender diverse (TGD) youth to gender-affirming medical care (GAMC) has become a contentious topic in the West, with many members of the general population, politicians, and even some experts and academic researchers voicing concerns about possible adverse effects of GAMC on the mental and physical health of TGD youth. Due to these concerns, recent years have seen a significant rise in legislation restricting TGD youth from accessing GAMC in countries such as the United States, the United Kingdom, and Canada. However, in this critical review of the literature on the psychological (e.g., anxiety, depression, suicide, and body satisfaction) and physical (e.g., bone health, cognitive function, and fertility) health outcomes associated with GAMC among TGD youth, we argue that, given the state of current research, youth should not be restricted from accessing GAMC. **Conclusions:** Our findings reinforce the importance of close monitoring by doctors, counselling for TGD youth with respect to potential risks, and increased studies on the topic, especially those focusing on reproductive health.

## 1. Introduction

Providing gender-affirming medical care (GAMC; i.e., medical care that works to support and affirm an individual’s gender identity) to transgender and gender diverse (TGD) youth (i.e., young people under the age of 18 whose gender differs from their sex assigned at birth) has become a contentious topic in the West. Despite an extensive and steadily growing body of research suggesting that GAMC for TGD youth is a safe and effective way to reduce gender dysphoria (i.e., distress due to incongruence between one’s experienced gender and one’s sex assigned at birth) [1] and associated mental health issues [2,3,4], members of the general population (e.g., 46% of American adults) [5], politicians (e.g., Canada’s Conservative party leader, Pierre Poilievre) [6], and even some experts and academic researchers (e.g., Abbruzzese et al. [7]; American College of Pediatricians [8]; Cass [9]) continue to voice concerns and/or oppose GAMC for TGD youth. For example, in a recent article published in the *Journal of Sex & Marital Therapy*, Abbruzzese and colleagues [7] argued that “the field has a penchant for exaggerating what is known about the benefits of the practice [GAMC], while downplaying the serious health risks and uncertainties” (p. 673).

As a result of this controversy, in recent years, there has been a significant rise in legislation restricting TGD youth from accessing GAMC [10]. Between March 2021 and August 2024 in the United States, 26 states implemented laws limiting or prohibiting access to GAMC, affecting approximately 40% of TGD youth ages 13 to 17 [11]. Similarly, in March 2024, England’s National Health Service [12] announced that puberty blockers (i.e., pharmaceutic interventions that delay the progression of puberty) would no longer be available for TGD people under the age of 18 due to “not enough evidence to support [their] safety or clinical effectiveness” (p. 3). (Puberty blockers, however, are available to youth attending select private clinics as well as those participating in clinical research trials.) Similar laws have been enacted in Canada. In Alberta, Premier Danielle Smith announced in February 2024 that the provincial government would introduce several new policies regarding TGD persons later in the year, including those restricting access to GAMC for youth [13]. Despite receiving extensive pushback from several legal and health organizations—including the Canadian Human Rights Commission [14], Canadian Paediatric Society [15], Canadian Psychological Association [16], and the Canadian Medical Association [17]—the Alberta government introduced and passed the *Health Statutes Amendment Act* in late 2024, which prohibits anyone under the age of 18 from undergoing top or bottom surgery and anyone under 16 from receiving puberty blockers or hormone therapy (i.e., pharmaceutical interventions that promote the development of physical features that are aligned with an individual’s gender identity) [18].

Those with concerns about GAMC for TGD youth cite the need to protect youth from making irreversible and harmful changes to their body and mental and physical health [7,9], while others argue that these concerns are largely misinformed [3]. In the current review, we aimed to address and evaluate these concerns through an up-to-date critical discussion of the health-related benefits and risks associated with GAMC for TGD youth. Additionally, although several recent reviews on the issue exist [19,20,21,22,23,24], these reviews tend to focus on a particular form of GAMC (e.g., hormone therapy) [2,19,20,21,22,23] and/or a specific aspect of health (e.g., fertility) [22,23,24]. Therefore, a secondary goal of this paper was to provide a comprehensive review of the mental and physical health benefits and risks of both hormone therapy and puberty suppression.

In the sections below, we provide a brief overview of mental health, minority stress, gender dysphoria, and GAMC among TGD youth. Then, after outlining our search methods and situating ourselves as researchers, we give an in-depth discussion of GAMC and its associated psychological and physical health risks and benefits among TGD youth. Finally, we critically summarize the main findings and associated implications and future directions of this paper.

### 1.1. Mental Health, Minority Stress, and Gender Dysphoria in TGD Youth

Compared to their cisgender counterparts, TGD youth experience significantly poorer mental health [25,26]. In a recent critical review of the literature, Wittlin and colleagues [26] noted that, compared to heterosexual and sexual minority cisgender youth, transgender youth are at increased risk of depression and anxiety, suicidality and self-harm, disordered eating (e.g., anorexia nervosa), and problematic substance use (e.g., alcohol, marijuana, and cigarettes). For these comparisons, all effect sizes and risk ratios were large. TGD youth are also overrepresented among homeless youth and in the foster care system [27]. Notably, these mental health disparities have been documented in several countries across the West, including the United States [28,29,30], Canada [31,32], New Zealand [33], Australia [34,35], the United Kingdom [31,36], and the Netherlands [31]. These mental health disparities can largely be attributed to experiences of minority stress (i.e., stress related to being a member of a stigmatized group) [37,38] and gender dysphoria, both of which are discussed below.

#### 1.1.1. Minority Stress

According to minority stress theory, TGD youth experience unique distal/external (e.g., discrimination and family rejection) and proximal/internal (e.g., internalized stigma) stressors related to their gender identity that cisgender youth typically do not experience [37,38]. These minority stressors can have adverse impacts on the mental health of TGD youth. For example, in a study of 1943 TGD adolescents (aged 14–18) from the United States, Jardas and colleagues [39] used structural equation modeling to examine associations between minority stress and mental health; they also assessed how these associations differed based on gender identity and race/ethnicity. The researchers found that experiences of prejudice, expectations of rejection, internalized stigma, and gender identity concealment were directly associated with higher levels of depression and/or anxiety symptoms. The researchers also found that, in general, transfeminine and transmasculine youth reported significantly more minority stress and mental health issues compared to nonbinary youth. In contrast, apart from Black TGD youth who reported significantly less minority stress and mental health issues than White TGD youth, findings were relatively consistent across racial/ethnic identities (i.e., White versus Latinx, Asian, multiracial, etc.). Relatedly, in a systematic review of 44 articles assessing risk factors for mental health issues among TGD youth, Tankersley and colleagues [40] identified poor peer relations (e.g., experiences of bullying); victimization, discrimination, and abuse; and, to a lesser extent, internalized stigma as common risk factors. Common risk factors not directly related to gender minority stress included older age, weight dissatisfaction, and low self-esteem.

The current sociopolitical climate in the West, which is rife with harmful misinformation about TGD people and threats to the accessibility of GAMC, has become an increasingly prominent distal minority stressor for TGD youth [3]. Highlighting this, Cunningham et al. [41] looked at the relationship between anti-TGD laws introduced between July 2019 and July 2020 in the United States and suicide- and depression-related Google searches. The researchers found that when a state passed anti-TGD legislation, Google searches related to depression increased by about 5% and searches related to suicide increased by 13 to 17% within that state. In contrast, when an anti-TGD state bill was defeated, the frequency of depression-related searches decreased by about 1 to 2%, though searches related to suicide did not change significantly. In another American study, Lee and colleagues [42] assessed the causal effects of state-level anti-TGD laws on suicide risk among a sample of more than 61,000 American TGD young people aged 13 to 24 across 34 states. With data collected across five time points between March 2018 and December 2022, the authors found that living in a state that had enacted anti-TGD laws during the study period was associated with a 38 to 44% increase in the number of past-year suicide attempts (also see Abreu et al. [43]; Dhanani & Totton [44]).

#### 1.1.2. Gender Dysphoria

In addition to minority stress, gender dysphoria (which is sometimes conceptualized as a form of proximal minority stress [45,46]) can contribute to mental health disparities among TGD youth, especially those who are post-pubescent. Before youth reach puberty (which typically begins between the ages of eight and 13 in youth assigned female and between nine and 14 in those assigned male) [47], their bodies are relatively similar regardless of sex assigned at birth [26,48]. However, gender dysphoria among youth can emerge or intensify at the onset of puberty, when their body begins to change in accordance with their assigned sex [26]. For many youths, the longer puberty progresses, the more distress they experience [49], with some youth describing gender-incongruent puberty as feeling like a betrayal [26,50].

While the Diagnostic and Statistical Manual of Mental Disorders (DSM-5) [51] considers gender dysphoria to be a mental health condition, gender dysphoria also has been shown to influence other aspects of TGD individuals’ mental wellbeing [46,52,53]. For instance, with a sample of 239 TGD young adults (aged 18–29) in the United States, Pease and colleagues [53] found that, along with distal minority stress (e.g., discrimination and non-affirmation of gender identity), gender dysphoria was directly associated with psychological distress. The researchers also found that gender dysphoria mediated the relationship between distal stress and psychological distress, suggesting that when TGD individuals experience distal stress, feelings of gender dysphoria increase, which subsequently contribute to greater feelings of psychological distress. Similarly, in a study of 109 TGD adolescents (aged 12–18) attending a medical center in the United States, participants who reported greater gender incongruence (i.e., the source of gender dysphoria) were more likely to meet diagnostic criteria for major depressive disorder than participants who reported lower gender incongruence [52].

The association between gender dysphoria and worsened mental health is further evidenced by research showing that gender-affirmation (i.e., the process whereby a TGD person’s gender identity is affirmed through social, medical, legal, and/or other means) not only serves to alleviate gender dysphoria but also improves other aspects of one’s mental wellbeing [46]. Gender-affirmation has been shown to alleviate symptoms of depression [54,55], anxiety [55,56], suicidality [55], and self-harm [56]. For example, among a sample of 73 prepubescent TGD youth and 49 of their cisgender siblings (aged 3–12), Olson et al. [54] found that when families supported their TGD child to socially transition (e.g., change their name and pronouns in accordance with their gender identity) and live as their authentic selves, TGD youth experienced rates of depression and anxiety comparable to their cisgender siblings. The authors also noted that the TGD youth who socially transitioned experienced significantly lower rates of depression and anxiety than previously reported rates among TGD youth living as their sex assigned at birth [57]. These findings suggest that some mental health issues are secondary to gender dysphoria [58] and, therefore, when treating gender dysphoria, there may be a subsequent reduction in mental health issues [46].

### 1.2. Gender-Affirming Medical Care for TGD Youth

The treatment of gender dysphoria in TGD youth is complex and individualized. Depending on various factors, including a youth’s age and individual experience of gender dysphoria, a range of options can help alleviate aspects of gender dysphoria, such as mental health interventions, parental support interventions, and social transition processes [3]. However, psychosocial interventions alone often do not alleviate gender dysphoria in TGD youth, particularly in post-pubescent youth who have begun to experience bodily changes in accordance with their assigned sex [3]. As such, many TGD youth seek out GAMC to supplement other forms of treatment.

There are two broad categories of GAMC: nonsurgical options, such as puberty blockers and hormone therapy, and surgical options, such as top surgeries (i.e., chest feminization and masculinization surgeries), bottom surgeries (e.g., vaginoplasty or phalloplasty), and facial reconstructive surgery [1]. While GAMC options available to TGD youth in the West vary considerably from country to country (and state to state in places like the United States), in Canada, all forms of GAMC are currently legal and available to TGD youth; the only exception is bottom surgery, which is restricted to those 18 years of age or older [1], as recommended by the World Professional Association for Transgender Health’s (WPATH) standards of care [59] and the Endocrine Society’s clinical practice guidelines [60]. (As noted earlier, due to implementation of its *Health Statutes Amendment Act*, this no longer applies to Alberta [18].) Further, as recommended by these organizations [59,60], top surgery is generally only performed in rare cases on adolescents who are at least 16 years of age and when they have received care for a significant duration of time [1]. For example, demonstrating this rarity, 223 chest surgeries were performed on people under 18 in Alberta between January 2022 and February 2023; however, only 8 (3.6%) of these surgeries were for treatment of gender dysphoria [61]. The remainder were performed for other medical reasons (e.g., cancer).

The most common forms of GAMC sought out by and offered to TGD youth are puberty blockers and hormone therapy, both of which are only recommended to youth after the onset of puberty [1,59,60]. Puberty blockers, or gonadotropin-releasing hormone agonists (GnRHa), are used to temporarily pause puberty from progressing and work by blocking hormones (i.e., testosterone and estrogen) that lead to puberty-related changes in the body [1]. While puberty blockers are also used among cisgender youth presenting with precocious puberty [47], in TGD youth, the goal is to “provide a young person with time to further explore their gender identity without pressure or distress related to ongoing development of secondary sex characteristics, or gendered experiences such as menses or erections. Should a young person continue to express gender dysphoria over time and eventually wish to pursue other gender-affirming treatments, [puberty blockers] may also prevent the further development of irreversible secondary sex characteristics that can make medical and surgical transition more difficult. Additionally, their blocking action may also allow for the use of lower doses of gender-affirming hormones to achieve phenotypic transition goals later on” [1] (‘Hormone Blockers’ section, para. 3).

Importantly, while some concern about reversibility exists [9,22], the current consensus among professionals in the field is that the effects of puberty blockers are reversible [1,59,60]. In other words, should youth decide not to continue with GAMC, puberty and its associated bodily changes will resume once puberty blockers are discontinued [1]. According to WPATH, puberty blockers can safely be taken for up to four years [59].

In addition to puberty blockers, some TGD youth seek out gender-affirming hormone therapy. Hormone therapy, which involves administering exogenous endocrine agents, is prescribed to promote the development of physical features that are better aligned with a TGD person’s gender identity [23]. TGD individuals assigned female wishing to appear more masculine are prescribed testosterone, while those assigned male wishing to appear more feminine are prescribed estrogen and anti-androgens [23]. While the effects of puberty blockers are considered fully reversible, hormone therapy is considered partially reversible, meaning some changes are reversible while others are not [1]. Specifically, for TGD individuals assigned female, reversible effects include menstrual suppression, increased libido, fat redistribution, and increased muscle mass, while effects such as voice deepening, clitoral enlargement, and body and facial hair growth are considered irreversible. For those assigned male, fewer spontaneous erections, fat redistribution, decreased muscle mass, and changes in body hair quality are considered reversible; breast tissue development is not. According to WPATH, hormone therapy is typically maintained throughout life, though some TGD people choose to discontinue once they have reached their desired changes [59].

For many (though not all) TGD youth, GAMC is considered medically necessary in treating gender dysphoria [62]. However, due to rising controversy surrounding its safety and effectiveness, as well as ongoing discussion about whether TGD minors can provide informed consent for treatment [2,7], TGD youth across the West are at increased risk of losing access to puberty blockers and hormone therapy through legislative restrictions. For example, of the 26 American states that have limited TGD youth from accessing GAMC in the last four years, 24 of them include bans on puberty blockers and hormone therapy (in addition to gender-affirming surgeries) until the age of 18 (or 19 in two states: Alabama and Nebraska) [11]. Similarly, while hormone therapy is available to youth in the United Kingdom, puberty blockers were banned for youth under the age of 18 in March 2024 [12]. With the goal of evaluating concerns about the safety of these interventions for TGD youth, general trends within research assessing mental and physical health-related risks and benefits of GAMC for TGD youth are discussed below. As noted above, puberty blockers and hormone therapy are the most common interventions offered to TGD youth and, therefore, are the focus of our discussion.

## 2. Methods

Due to the broad scope of this review, we approached this paper as a critical review, which is a non-systematic type of narrative review that is flexible, interpretative, and often involves a critical point of view [63]. In line with Sukhera’s [64] guidelines for narrative reviews in medical education, as well as recently published reviews pertaining to LGBTQ (lesbian, gay, bisexual, transgender, queer) health [65,66], our search strategy involved the following. Literature was located through PubMed, PsycINFO, and Google Scholar between December 2024 and June 2025. PubMed and PsycINFO were selected as they provide coverage of the medical and psychological literature, respectively, and Google Scholar—a search engine covering a broad scope of disciplines and topics—was selected with the goal of locating literature that may be missed through PubMed and PsycINFO. Each database was searched using terms related to TGD youth (e.g., “trans,” “transgender” and “youth,” “adolescents”), GAMC (e.g., “gender-affirming medical care”, “puberty blockers”, “hormone therapy”), psychological health outcomes (e.g., “mental health,” “anxiety,” “body satisfaction”), and physical health outcomes (e.g., “physical health,” “bone health,” “fertility”). This process was iterative and involved several cycles of searches using as many variations of each term as possible to ensure sufficient coverage of the literature [63]. Additional literature was located by searching within the selected articles’ references. We also searched selected articles’ citation lists using Google Scholar to ensure we had the most up-to-date studies on each topic (e.g., influence of puberty blockers on fertility). Finally, the peer-review process itself was valuable in broadening our search.

Abstracts of retrieved articles were manually reviewed and evaluated for inclusion based on several inclusion/exclusion criteria. Specifically, original quantitative studies and reviews of quantitative research findings were considered for inclusion if they were (1) directly related to health outcomes associated with the use of puberty blockers and/or hormone therapy; (2) on human participants (rather than animals); (3) peer-reviewed; (4) available in English; and (5) published in the last 25 years (i.e., between 2000 and 2025). Therefore, studies were excluded if they were unrelated to the review’s topic, exclusively qualitative, opinion pieces, editorials, not peer-reviewed, or unavailable in English. Using a similar rationale as Winters and associates [67], we excluded studies that were published before 2000, as “The language of self-identification with respect to gender identity, gender dysphoria, and transgender social identity was not available to children then [e.g., 1980s], as it is today” (p. 247). Studies involving animals (e.g., rats) instead of humans were excluded. We also did not consider master’s theses, doctoral dissertations, grey literature (e.g., policy reports), grey data (e.g., blogs), or grey information (e.g., emails). (For details about these categories of grey knowledge, see Adams and colleagues [68].) We did not exclude articles based on their findings; that is, articles were included regardless of whether they reported positive health outcomes, negative health outcomes, or null outcomes.

As an illustrative example, in PsycINFO, 11,449 articles contained the key words “gender non-conforming,” “gender diversity” and “transgender.” By restricting this pool to articles that were “peer-reviewed,” “published in English,” and published within “2000–2025,” 8408 articles were retained. The term “puberty blockers” was then entered as a new search term, resulting in 2528 articles. The Boolean operator “and” was used between the 8408 and 2528 articles, resulting in 228 articles (i.e., these articles were peer-reviewed, published in English, published during the targeted time frame, and concerned puberty blockers). We then used the Boolean “and” operator between the 228 articles and separate keyword listings for children (630,248) and adolescents (220,139), resulting in 20 articles focusing on children and puberty blockers and 40 articles concerning adolescents and puberty blockers. Each of these articles was then reviewed manually for possible inclusion. This search process was repeated for all topics of interest (i.e., psychological health outcomes, physical health outcomes, etc.).

### Researchers’ Positionalities

Members of this authorial group include heterosexual, gay, cisgender, and gender non-conforming persons of various ages, who occupy different roles within academia (e.g., graduate student, professor, and instructor). Some of us are parents, married, divorced, and/or single. Thus, as a group, we are socio-demographically diverse. We are, however, similar in our beliefs that (a) gender is fluid, evolves, and may or may not be binary; and (b) ethical research with TGD persons is rooted in awareness that any gender identity outcome (e.g., trans, cisgender, nonbinary, etc.) is equally “acceptable, healthy, and valid” [69] (p. 447). Of course, such beliefs do not preclude our team’s ability to conduct an unbiased critical review of the published literature on TGD youth and GAMC. As Larregue [70] observes, there is little evidence to suggest that scholars with a liberal viewpoint on, say, gender, are necessarily less impartial or more prone to embrace advocacy in the scientific knowledge they produce than their more conservative counterparts.

## 3. Results

### 3.1. Mental Health Outcomes

Below, we review studies assessing the associations between GAMC (i.e., puberty blockers and hormone therapy) and various mental health factors (i.e., depression, anxiety, suicidality, self-harm, gender dysphoria, body satisfaction, etc.) among TGD youth. These factors were selected for review because, after an initial inspection of the literature, they appeared to be discussed most frequently. In general, the findings of these studies suggest GAMC is associated with improved mental health among TGD youth.

#### 3.1.1. Depression and Anxiety

Overall, researchers indicate that, among TGD youth, use of puberty blockers is associated with decreased depression [55,71,72,73,74,75,76,77] and, albeit less consistently, anxiety [55,71,72,73,74,76,77]. Highlighting this, in a retrospective cohort study of 438 American TGD youth between the ages of 13 and 17, McGregor et al. [74] compared the mental health of participants who were (*n* = 40) and who were not (*n* = 398) treated with puberty blockers. With data collected during a routine clinical assessment for hormone readiness, the authors found that youth taking puberty blockers (for an average of 18 months) had significantly lower scores for anxiety (*d* = −0.64) and depression problems (*d* = −0.76) than youth not taking puberty blockers. These associations remained statistically significant when gender was controlled. In contrast, in a prospective follow-up study of 70 TGD adolescents (mean age at assessment = 13.6) attending the Amsterdam gender identity clinic in the Netherlands, de Vries et al. [72] assessed changes in participants’ mental health between the start of treatment and before the start of hormone therapy (about two years later, on average). The researchers found that, while scores for depression significantly decreased (*p* = 0.004, *d* = −0.48), the observed decrease in anxiety scores was neither statistically (*p* = 0.276) nor practically (*d* = −0.17) significant. Taken together, while the use of puberty blockers appears to be associated with decreased depression in TGD youth, the association between puberty blockers and anxiety is less consistent, with some studies reporting decreased anxiety [70,72,73,76] and others reporting null findings [55,72,77]. Future research is needed to determine the source of this inconsistency.

Most studies assessing changes in depression [55,76,78,79,80,81,82,83,84,85,86] and anxiety [55,79,80,81,84,85,86] following hormone therapy have documented reductions in symptomology. In a prospective cohort study that followed 315 TGD youth (aged 12 to 20) for two years after hormone therapy initiation in the United States, Chen and colleagues [80] found that depression and anxiety significantly decreased during the study period (*d*s = −0.20 and 0.25, respectively). The authors also compared youth who had initiated gender-affirming hormones (GAH) during early puberty with youth who had not done so (i.e., initiated GAH in late puberty) and found that the early group reported lower levels of depression (*d* = 0.70) and anxiety (*d* = 0.79) than their late-puberty counterparts, suggesting beginning hormone therapy earlier was associated with better outcomes. Similarly, in an American study comparing the mental wellbeing of 19 adolescent transgender boys receiving testosterone for approximately 13 months to 23 adolescent transgender boys not receiving treatment, generalized anxiety (*d* = −0.86), social anxiety (*d* = −1.37) and depression (*d* = −0.86) severity were significantly lower among the former group when controlling for age [81].

#### 3.1.2. Suicidality and Self-Harm

Many studies also have examined whether puberty blocker treatment is associated with lower rates of suicidality (e.g., suicidal thoughts, suicidal ideation) [4,73,74,75,87] and self-harm [75,88,89]. Of these studies, all but one (which reported null findings with respect to self-harm) [89] reported that puberty blockers are associated with significantly lower rates of suicidality and self-harm. For example, with a sample of 272 TGD adolescents who had not received care (mean age = 16.75), 178 TGD adolescents taking puberty blockers (mean age = 14.47), and 651 cisgender adolescents (mean age = 15.39), Dutch researchers van der Miesen et al. [75] found that TGD adolescents treated with puberty blockers reported significantly less suicidality and self-harm (assessed as a single variable) than TGD adolescents who had not received treatment (*d* = −0.36). The researchers also noted that, while the untreated TGD group reported significantly higher suicidality and self-harm than the cisgender comparison group (*d* = 0.32), the TGD adolescents who were treated with puberty blockers did not differ significantly from the cisgender group (*d* = 0.04), suggesting puberty blockers improved their mental wellbeing to levels typically seen in the cisgender population. Finally, the authors also noted that findings remained similar when statistically controlling age, ethnicity, education level, and parents’ marital status.

With one known exception reporting null findings [86], most studies assessing the influence of hormone therapy on suicidality [55,76,83,86,90,91,92,93] and self-harm [55,91] have reported positive findings. For instance, using data collected from 11,914 American TGD young people (aged 13 to 24), Green and colleagues [83] compared the mental wellbeing of TGD youth who had received hormone therapy to those who wanted hormone therapy but had not yet received it. After controlling for demographic covariates (e.g., age, socioeconomic status, and race), as well as parental support, experiences of victimization, receipt of puberty blockers, and exposure to gender identity conversion therapy, the authors found that, while the adjusted odds ratio (aOR = 0.73) for suicide attempts did not reach statistical significance (*p* = 0.16), participants who had received hormone therapy had significantly lower odds of seriously considering suicide than participants who had not received hormone therapy (aOR = 0.74, *p* < 0.001). Similarly, in a retrospective chart review of 52 TGD adolescents (aged 15–20) attending a gender identity clinic in Finland, Kaltiala et al. [91] assessed the relationship between hormone therapy and the need for treatment related to suicidality and self-harm. By comparing data from participants’ initial assessment to data collected one year after beginning treatment, the researchers found that the proportion of participants needing treatment related to suicidality and self-harm (assessed as a single variable) decreased significantly, from 35% at assessment to 4% one year later (*p* < 0.001).

#### 3.1.3. Gender Dysphoria and Body Satisfaction

In terms of gender dysphoria and other related constructs, such as body satisfaction and body image, puberty blockers appear to have little influence [71,72,73,89]. One exception is Fisher et al.’s [73] prospective study of 36 TGD adolescents (aged 11–15) from Italy, wherein participants reported significant improvements in body uneasiness (i.e., body-related psychopathology, including weight dissatisfaction, compulsive behaviours, and worries about certain body parts) after at least three months of GnRHa therapy, *d* = 0.34. In contrast, in a longitudinal study of 55 young transgender adults (aged 20–23 at the end of the study) who received GAMC (puberty blockers, hormone therapy, and gender reassignment surgery) in the Netherlands, de Vries et al. [71] noted significant improvements in body image (*d* > 0.80) and gender dysphoria (*d* > 0.80) following hormone therapy and/or gender reassignment surgery, but not puberty blockers. However, this finding makes sense because, as noted earlier, the primary goal of puberty blockers is to provide TGD youth time to explore their gender identity without pressure or distress related to the ongoing development of secondary sex characteristics that are inconsistent with their experienced gender [1]. Puberty blockers do not change a youth’s body in the desired direction and, therefore, are unlikely to contribute to significant improvements in body image or gender dysphoria. Instead, these improvements tend to come later, with gender-affirming procedures like hormone therapy and/or gender reassignment surgery [89], as they did in de Vries et al.’s [71] study.

In line with de Vries et al. [71], researchers have shown consistently that hormone therapy is associated with improvements in gender dysphoria [85,92] and similar constructs like body satisfaction [79,80,81,82,84,92,94]. To illustrate, in a prospective study of 23 TGD patients (aged 14–18) receiving hormone therapy at an endocrinology clinic in Spain, Lopez de Lara and colleagues [85] found that gender dysphoria severity—as measured by the Gender Dysphoria—Utrecht Scale wherein a score greater than 40 points indicates gender dysphoria—decreased significantly after one year of treatment, from a mean score of 57.1 before hormone therapy to a mean score of 14.7, 12 months into treatment (*p* < 0.001). In another study, Kuper et al. [84] assessed the mental wellbeing of 148 TGD youth receiving hormone therapy in the United States. By comparing data collected at initial assessment to data collected approximately one year later, the researchers found that participants’ body dissatisfaction had significantly decreased (*d* = 1.04).

#### 3.1.4. Additional Mental Health Outcomes

Researchers also have assessed the associations between the use of puberty blockers, hormone therapy, and general mental health factors such as overall quality of life, psychological distress, psychosocial functioning, and subjective wellbeing [71,72,79,80,90,95,96,97]. In general, this research has shown positive improvements in these factors following the use of puberty blockers among TGD youth. For example, in de Vries et al.’s [72] prospective follow-up study of 70 TGD adolescents (mean age at assessment = 13.6) attending the Amsterdam gender identity clinic in the Netherlands, the authors found that global functioning scores had significantly improved after about two years of puberty suppression, *d* = 0.48.

In another study using data from the 2015 US Transgender Survey, Lee and colleagues [96] compared health outcomes of TGD adults who had received puberty suppression and/or hormone therapy during adolescence (before age 18) compared to those who did not. The researchers found that, whereas 52% of participants who wanted but did not receive puberty blockers reported severe psychological distress, only 34% of those who had received it reported psychological distress (*p* < 0.001). Similarly, while 43% of those who wanted but did not receive hormone therapy reported severe psychological distress, only 38% of those who did receive hormone therapy reported distress (*p* < 0.003).

Finally, in Chen et al.’s [80] American prospective cohort study that followed 315 TGD youth (aged 12 to 20) for two years after hormone therapy initiation, the authors found that life satisfaction significantly increased during the study period (*d* = −0.39). They also found that, when comparing youth that had initiated gender-affirming hormones (GAHs) during early puberty with youth that had not done so (i.e., initiated GAHs in late puberty), the early group reported significantly greater levels of positive affect (*d* = 0.69) and life satisfaction (*d* = 0.45) than their late-puberty counterparts, suggesting beginning hormone therapy earlier was associated with better outcomes.

### 3.2. Physical Health Outcomes

In addition to mental health, concerns have been raised about the short-term and long-term impacts of puberty blockers and hormone therapy with TGD youth on their physical health. As such, the potential impacts on several areas of concern, including bone health, body composition, cardiometabolic health, cognitive function, and fertility, are discussed below. These factors were selected for review because, after an initial inspection of the literature, they appeared to be discussed most.

#### 3.2.1. Bone Health

One of the main physical health concerns regarding GAMC for TGD youth pertains to bone health. Puberty is a key period for bone development, with approximately 40 to 60% of adult bone mass being accrued during this time [98]. As such, puberty blockers, which block puberty from progressing, can have short-term negative impacts on bone development or bone density in TGD youth, especially in the lumbar spine (i.e., the vertebra in the lower back), which may subsequently lead to other adverse outcomes, such as increased risk of fractures [65,99,100,101,102,103]. For example, in a retrospective study of 46 Belgian TGD adolescents (mean age = 16) who took GnRHa for at least two years, Ciancia et al. [100] examined the effects of puberty blockers on bone mass acquisition using X-ray absorptiometry (DXA). The researchers found that, between the start of GnRHa initiation and before starting hormone therapy, bone mineral apparent density scores (i.e., a measure of bone density that considers body size) at the lumbar spine decreased significantly in both transgender boys and girls and significantly decreased at the femoral neck (i.e., part of the thigh bone located in the hip) in transgender boys but not girls. Similar findings have been reported elsewhere [101,102,103].

It should be noted, however, that researchers suggest decreased bone density while taking puberty blockers is fully or partially restored once puberty blockers are discontinued [104] and/or after the administration of hormone therapy (which, on its own, is not associated with adverse impacts on bone health) [65], though this may be more true for youth assigned female than those assigned male [102,103,104,105,106,107,108]. Highlighting this point, van der Loos and colleagues [106] assessed the long-term impacts of puberty suppression during adolescence on bone health among a sample of 75 TGD individuals (median age at long-term follow-up = 28) who had undergone at least 9 years of hormone therapy following the use of puberty blockers. Using data from a Dutch gender clinic, the researchers found that, after long-term hormone therapy, participants’ bone mineral density caught up to pre-treatment (of puberty blockers) levels at the lumbar spine, total hip, and femoral neck in those assigned female but only at the total hip and femoral neck in those assigned male. Based on these findings, the authors concluded that the use of puberty blockers followed by long-term hormone therapy is safe with respect to bone health, though those assigned male taking estrogen may require closer monitoring by clinicians than those assigned female taking testosterone.

Additional evidence comes from research conducted among cisgender women treated with puberty blockers during their youth for precocious puberty. In an Italian retrospective chart review study, 87 women who were treated with GnRHa for an average of 4.2 years during their youth were followed for approximately 10 years after discontinuation of treatment to assess the impact of GnRHa on adult bone heath [104]. The researchers found that bone mineral density reached normal levels upon complete resumption of gonadal activity. These initial findings are promising, though there is a need for more research on youth assigned male to better ascertain the long-term effects of puberty blockers on their adult bone health.

#### 3.2.2. Body Composition

Some researchers also have noted changes in body composition following puberty blocker intervention among TGD youth [105,109,110]. Specifically, findings from short-term studies have shown consistently that height SDS (i.e., a measure of how different a child’s height is from the average height for their age and sex) decreases during GnRHa treatment, especially during the first two years [89,103,111]. Short-term studies also have found that puberty blockers contribute to significant increases in body fat and decreases in lean body mass [109,111,112].

However, much like bone health, long-term research suggests that these changes in body composition typically correct themselves once puberty blockers are discontinued and/or through hormone therapy [109,110,112,113]. With respect to height, Ciancia and colleagues [113] conducted a retrospective study to assess the effects of puberty blockers and hormone therapy during adolescence on final height. With a sample of 32 TGD adolescents (mean age at final height = 18) who attended a pediatric health service in Belgium, the researchers found that while growth acceleration tended to decrease while taking puberty blockers, growth acceleration later increased during hormone therapy. As a result, participants’ final height did not significantly differ from the predicted adult height of their sex assigned at birth. On the other hand, some studies have reported that short-term treatment with puberty blockers followed by long-term treatment with hormone therapy can lead to adult height reductions in TGD individuals assigned male [114,115]. One should bear in mind, however, that such reductions in height are not inherently negative or harmful, and this may be a desired outcome for TGD girls given that they would achieve a height more closely in line with cisgender women [115].

In terms of body fat and lean body mass, findings are more mixed due to the differential effects of masculinizing and feminizing hormones [105,107,116]. In a retrospective study following 548 TGD adolescents (mean age at study onset = 14) attending a gender identity clinic in the Netherlands for three years, Boogers et al. [107] assessed the effects of both puberty blockers and hormone therapy on body composition. The researchers found that the use of puberty blockers was associated with increased fat mass and decreased lean mass in both transgender boys and girls. However, after the initiation of testosterone (which suppresses fat gain and promotes muscle growth), inverse effects were observed in transgender boys, suggesting that hormone therapy reversed the effects of puberty blockers. Although inverse effects were not observed among transgender girls, changes in their fat mass and lean mass did stabilize after initiating estradiol (which tends to promote fat gain and suppress muscle growth). Put simply, these findings suggest that puberty blockers and hormone therapy do not tend to have significant or permanent adverse impacts on TGD youths’ fat or muscle mass. Even when changes in fat and muscle mass do occur, these changes are not necessarily negative, though clinicians should discuss the possibility of such changes with TGD youth prior to initiating GAMC, and more long-term research is needed to confirm these findings.

#### 3.2.3. Cardiometabolic Health

Some researchers have studied the effects of puberty blockers and hormone therapy on aspects of TGD youths’ cardiometabolic health, including body mass index (BMI), cholesterol markers, blood pressure, and markers of diabetes [19,20]. With respect to puberty blockers, most studies have reported no effects on BMI [101,102,103,117,118], blood pressure [118,119,120], or markers of diabetes [121,122]. Hormone therapy during adolescence also has generally been shown to have little to no effect on these factors [20,118,121,123,124,125,126,127,128].

Research findings concerning the effects of puberty blockers and hormone therapy on cholesterol markers have been mixed [19,20]. Highlighting this, Taylor et al. [19] noted in their systematic review that three studies had assessed the relationship between puberty blockers and cholesterol (high-density lipoprotein [HDL] and low-density lipoprotein [LDL]). Of these, one study found no changes in HDL or LDL following initiation of GnRHa [118]; another study found decreased HDL but no change in LDL after initiating cyproterone acetate (i.e., an alternative to GnRHa) [122]; and the other study found decreased HDL and increased LDL following lynestrenol (i.e., another alternative to GnRHa) [121]. However, Tack et al. [122] did not regard the decrease in HDL to be “clinically relevant or [to be the] cause of clinical problems” (p. 751). The changes in HDL and LDL in Tack et al.’s study [121], while clinically relevant, did not necessitate stopping treatment.

Findings regarding hormone therapy and cholesterol markers among TGD youth are also mixed [20]. In Taylor and colleagues’ [20] systematic review, seven pre–post studies assessed HDL, with three reporting a clinically significant decrease [118,123,124], one a clinically non-significant increase [129], and three no change [121,122,123,130]. All three studies, which reported a decrease in HDL, did so in relation to testosterone treatment; no studies reported needing to discontinue a patient’s treatment because of changes in HDL. Finally, no studies reported changes in HDL following estrogen treatment.

#### 3.2.4. Cognitive Function

Due to the numerous changes in brain function and structure that occur during puberty (e.g., the maturation of the prefrontal cortex, which is primarily responsible for executive functions), some researchers have expressed concern over potential adverse impacts of GAMC—and puberty blockers in particular—on the cognitive function of TGD youth [22,131]. Despite such concerns, most researchers assessing the influence of puberty blockers on cognitive function have reported no clinically significant adverse effects [131,132,133,134,135,136,137]. For example, a Belgian study compared the cognitive function of 15 cisgender girls (median age = 10) undergoing GnRHa treatment for precocious puberty (treatment duration = 8 to 57 months) and 15 age-matched controls [136]. Findings indicated no significant group differences in IQ scores, memory, or executive function.

More recently, in a study of 72 TGD adolescents in the Netherlands, Arnoldussen and colleagues [132] assessed the association between IQ at study entry (mean age = 13) and educational achievement in early adulthood after treatment with puberty suppression and hormone therapy (mean age = 20). The researchers hypothesized that, because past research has demonstrated that IQ scores in childhood are positively associated with educational achievement later in life [138], a positive association between pre-treatment IQ scores and educational achievement post-treatment would provide a proxy for the potential effects puberty suppression (and hormone therapy) has on cognitive development. Indeed, the researchers found a significant positive relationship between pre-treatment IQ and educational achievement; they also noted that the correlation coefficient (Nagelkerke R = 0.71) was akin to that found in the general population, suggesting that puberty suppression (and hormone therapy) did not adversely affect cognitive function. Overall, these findings suggest that puberty suppression during adolescence does not have adverse effects on cognitive function. However, as noted by Baxendale [22], future research assessing the long-term impact of puberty suppression on cognitive function with larger samples is needed.

Studies assessing the relationship between hormone therapy and cognitive function have generally found no significant adverse effects [132,137,139,140]. In the Netherlands, Burke and colleagues [139] compared visuospatial functioning of 21 girls with GD (mean age = 16 years) receiving testosterone to two age-matched control groups (21 girls and 20 boys). The authors found no significant differences between groups on the mental rotation task (*d*s ranged from −0.29 to −0.02), suggesting that testosterone did not impair cognitive function. Additionally, in a meta-analytic review of ten studies assessing the impacts of hormone therapy on cognitive function in TGD young adults, Karalexi et al. [140] noted no significant adverse effects.

#### 3.2.5. Fertility

Some researchers have expressed concerns about the potential adverse impacts on fertility when GAMC occurs during adolescence. While research in this area is limited, the available literature indicates that the impacts of GAMC on fertility vary depending on the type(s) of GAMC received (i.e., puberty suppression, hormone therapy, or both), the timing of treatment (i.e., before, during, or after pubertal development), and the assigned sex of the patient [141,142]. For TGD youth who undergo puberty suppression then later discontinue without initiating hormone therapy, long-term adverse effects on fertility appear limited for both sex-assigned females and males [143]. Highlighting this, Lazar et al. [144] assessed the reproductive health of 153 Israeli cisgender adult women aged 25 to 56 who had undergone puberty suppression treatment for precocious puberty during childhood. Findings indicated no significant differences between treated women and an untreated, age-matched control group with respect to spontaneous pregnancy achievement and pregnancy complications, suggesting that puberty suppression did not negatively affect reproductive health. Indeed, a higher instance of fertility-related difficulties was experienced by women that had untreated central precocious puberty (CPP). Similarly, Bertelloni and colleagues [145] assessed the endocrine and exocrine testicular function of nine Italian cisgender men (mean age = 17) treated with GnRHa during childhood for an average of six years. The data indicated normal testicular function, suggesting that GnRHa treatment is safe with respect to fertility.

Regarding the effects of hormone therapy (without previous GnRHa treatment), much of what is known comes from research conducted with TGD adults [142]. In studies of transgender women, researchers have observed that estrogen reduces sperm quality [146,147,148,149]. For example, in a study assessing sperm quality in semen samples provided for fertility preservation by 212 Swedish transgender women (aged 14–54), Rodriguez-Walberg et al. [149] found that sperm quality was significantly lower in participants who had undergone hormone therapy than those who had not. Research findings among transgender men are less consistent, though still suggest hormone therapy can adversely impact fertility [142]. In Nahata et al.’s [142] narrative review, the authors noted that of the eight case studies examining the ovaries of transgender men taking testosterone, four reported adverse effects (e.g., polycystic ovarian morphology) [150,151,152,153], three found minimal to no negative effects [154,155,156], and the other’s findings fell somewhere in between [157]. Additionally, two studies have assessed the effects of testosterone therapy on anti-mullerian hormone (AMH) levels; of these, one found a decrease in AMH levels [158] and the other reported no change [121].

Although hormone therapy may have adverse effects on the reproductive health of TGD individuals, it typically does not mean they are unable to conceive. Prior to beginning hormone therapy, TGD adolescents can pursue oocyte (egg) or sperm cryopreservation, and have been recorded successfully doing so [141,159]. Additionally, even after TGD individuals have initiated hormone therapy, both transgender men and women have the option to temporarily stop treatment to conceive [141]. For example, in a longitudinal study of nine transgender women (mean age = 26) from the Netherlands and Austria who had undergone hormone therapy for a median duration of 36 months, de Nie et al. [147] reported the recovery of viable sperm in all participants 3 (*n* = 6), 8 (*n* = 1), 10 (*n* = 1), and 17 (*n* = 1) months after pausing hormone treatment. Similarly, among an international sample of 41 TGD men (mean age = 28) who had experienced pregnancy, 25 reported using testosterone before becoming pregnant, and those who used testosterone before becoming pregnant did not significantly differ from participants who had not used testosterone in terms of birth outcomes or complications (e.g., hypertension) [160]. Interestingly, the authors also noted that while 20 of the 25 participants who took testosterone stopped treatment before becoming pregnant, 5 of them became pregnant while still using testosterone. Therefore, while hormone therapy may have adverse impacts on the reproductive potential of TGD individuals, many are still able to successfully conceive, sometimes even without stopping treatment. However, researchers also need to acknowledge that, for an unknown number of TGD persons, fertility and conception may not be particularly salient [161], and more research is needed to determine the extent to which these findings extend to TGD individuals who begin hormone therapy during adolescence.

Finally, many TGD youth taking puberty blockers choose to subsequently undergo hormone therapy without discontinuing GnRHa beforehand [162]. For TGD youth who initiate GnRHa in later stages of puberty, oocyte (egg) or sperm cryopreservation is an option much like it is for adults [163]. Conversely, because pubertal development is vital for sperm production and egg maturation, and mature gametes (i.e., sperm and eggs) are typically not present until later in puberty [164], youth who initiate GnRHa in the early stages of puberty may not have this option. Theoretically, they should be able to pause GAMC before or after switching from puberty blockers to hormone therapy to allow for innate puberty to progress enough to develop mature gametes should they wish to have biological children [142,165]. However, there is limited research testing this theory, and more research is needed to determine the options available to TGD individuals who undergo both puberty suppression and hormone therapy [142,143,144,145,146,147,148,149,150,151,152,153,154,155,156,157,158,159,160,161,162,163,164,165]. Until then, it is vital that clinicians ensure their patients and patients’ caregivers are aware of these risks.

## 4. Conclusions

As shown in this review, puberty blockers and hormone therapy are associated with a wide range of health outcomes among TGD youth. Overall, researchers suggest that puberty blockers and hormone therapy have positive implications for the mental health of TGD youth, including decreased depression, anxiety, suicidality, self-harm, gender dysphoria, and body dissatisfaction, as well as increased quality of life and life satisfaction. On the other hand, like most (if not all) medical interventions, GAMC also has some risks. These risks can include decreased bone density, changes in body density, reduced cardiometabolic health, and, perhaps most notably, fertility issues. However, these risks do not necessarily suggest a need to prohibit TGD youth from accessing GAMC [3], but rather underscore the need for close monitoring by doctors (e.g., regular psychological assessments, blood pressure tests, and bloodwork) [59], counselling for TGD youth with respect to potential risks, particularly in relation to fertility, and increased research on the topic [59,60]. Indeed, we noticed throughout our review researchers stressing the importance of collaborative care between TGD persons and an interdisciplinary team as well as supportive family and friends.

At the same time, the need for additional high-quality studies on GAMC for TGD youth cannot be overlooked. Although puberty blockers and hormone therapy have been used for over two decades, and the effects of these interventions have been well documented [166], there remain several important avenues for future research to better understand the benefits and risks associated with GAMC for TGD youth. First, much of the literature assessing the health outcomes associated with GAMC for TGD youth can be considered low-quality, relying primarily on small samples, correlational designs, and short-duration follow-ups [167,168,169]. Highlighting this, in a 2018 systematic review of health outcomes associated with puberty suppression and hormone therapy among TGD youth, Chew et al. [167] found that, of the 13 studies included in their review, all were associated with a medium to high risk of bias (according to the Quality of Prognosis Studies tool), most included small samples (ranging from 21 to 201), only two studies included controls, and no studies used blinding or randomization. More recently, in a systematic review of the same topic using the Grades of Recommendations, Assessment, Development, and Evaluation (GRADE) system, Tornese and colleagues [169] found that, of the 51 studies identified for their review, 22 were moderate-high in quality and the remaining 29 were low quality. Additionally, sample sizes ranged from 13 to 410, 19 studies were prospective longitudinal, 27 were retrospective, and 5 were cross-sectional, and the duration of follow-up ranged from 6 months to 22 years. Therefore, while quality appears to be improving, there remains significant room for improvement.

Some researchers have argued for randomized control trials (RTCs), because, according to them, without such trials, the documented benefits of GAMC may be the result of a placebo effect [8,170,171]. However, there is already extensive evidence that GAMC benefits TGD youth and, therefore, it would be unethical to conduct RTCs because “randomizing transgender people to receive or not receive hormone therapy or surgery violates the principle of equipoise, or true scientific uncertainty about whether an intervention will help the individual” [3] (p. 852). Instead, we encourage increased research using longitudinal designs, natural experiments, matched-control cohorts, and larger samples. Further, much of the research on the topic focuses on WEIRD (Western, Educated, Industrialized, Rich, and Democratic [172]) samples, and more research is needed on youth from different backgrounds (e.g., racial and ethnic minorities) to assess the extent to which the findings within the current review generalize beyond WEIRD samples. While additional research on all mental and physical health outcomes is encouraged, there is a particular need for more research on the reproductive health of TGD youth who undergo GAMC, especially those who undergo both puberty suppression and hormone therapy, as much of what is known is based on research with cisgender youth with precocious puberty or TGD adults, and may not generalize to TGD youth [142]. Such research is important and would not only allow clinicians to better inform patients of their reproductive options (e.g., cryopreservation), but also improve the outcomes associated with these options.

The findings of the present review should be interpreted with several methodological strengths and limitations in mind. Compared to systematic reviews, narrative reviews offer more flexibility, are especially advantageous for broad or complex topics requiring detailed description and interpretation and are useful when learning or teaching about a particular issue because they provide a general overview [63,64]. At the same time, narrative reviews do not typically provide an exhaustive review of all literature on a topic and, relatedly, are often criticized because they are selective and more subjective. They are also less replicable due to their non-systematic approach [63,64].

Our decision to exclude certain literature from our review (e.g., grey literature), while done to refine the scope of the review and ensure only higher quality, up-to-date research was included, also has limitations. For example, a key disadvantage to excluding grey literature, such as dissertations and policy reports, is publication bias (i.e., the tendency for peer-reviewed journals to overlook studies with null findings) [173]. However, our research question concerned the psychological and physical health outcomes of GAMC. Findings that do not support GAMC (i.e., findings that suggest GAMC may compromise psychological and physical health) are not null. Thus, unless one contends that commercial/academic publishing companies have a vested interest in promoting GAMC, it is unclear why peer-reviewed studies would disproportionately favor one side of the GAMC debate. Indeed, the publication of opinion pieces critical of GAMC in peer-reviewed outlets—see, for example, Evans [174]—suggest this is not the case.

Taken together, this review outlined the mental and physical health outcomes associated with puberty suppression and gender-affirming hormone therapy among TGD youth. While risks do exist, particularly when it comes to fertility, and ongoing research is inarguably needed, it remains difficult to justify a “wait and see” approach, given the temporal nature of pubertal development and the available information demonstrating the importance of GAMC in maintaining and improving mental wellbeing (e.g., reducing the risk of suicide) [41,42,43,73,74,75,90,91,92,93]. Our final question: How many TGD youths should be required to develop bodies they do not believe reflect who they are as researchers wait for that ever-elusive final study that will “prove” gender-affirmative care is salutary?
